# Magnitude of the Freshwater Turtle Exports from the US: Long Term Trends and Early Effects of Newly Implemented Harvest Management Regimes

**DOI:** 10.1371/journal.pone.0086478

**Published:** 2014-01-27

**Authors:** Ivana Mali, Michael W. Vandewege, Scott K. Davis, Michael R. J. Forstner

**Affiliations:** 1 Department of Biology, Texas State University, San Marcos, Texas, United States of America; 2 Department of Biochemistry, Molecular Biology, Entomology and Plant Pathology, Mississippi State University, Mississippi State, Mississippi, United States of America; 3 Turtle Survival Alliance, Fort Worth, Texas, United States of America; CSIR- National institute of oceanography, India

## Abstract

Unregulated commercial harvest remains a major threat for turtles across the globe. Due to continuing demand from Asian markets, a significant number of turtles are exported from the United States of America (US). Beginning in 2007, several southeastern states in the US implemented restrictions on the commercial harvest of turtles, in order to address the unsustainable take. We have summarized freshwater turtle exports from the US between 2002 and 2012 and demonstrated that the magnitude of turtle exports from the US remained high although the exports decreased throughout the decade. Louisiana and California were the major exporters. The majority of exports were captive bred, and from two genera, *Pseudemys* and *Trachemys*. We review the changes over the decade and speculate that the increase in export of wild turtles out of Louisiana after 2007 could be a consequence of strict regulations in surrounding states (e.g., Alabama, Florida). We suggest that if wild turtle protection is a goal for conservation efforts, then these states should work together to develop comprehensive regulation reforms pertaining to the harvest of wild turtles.

## Introduction

Turtles are a substantial commodity in the global market of wildlife commercial goods and sustenance. For example, in 1995 the estimated value of freshwater turtle exports from Vietnam to China exceeded 1 million US dollars [1]. Recent and historic over harvest led to severe declines and even subsequent extinctions of several freshwater turtle, sea turtle, and tortoise species [2], [3]. Today, chelonians represent one of the most endangered taxonomic groups on the planet [4]. China is the world’s largest consumer of turtles, where meat and shells are believed to have medicinal value [5], [6], [7]. High demands from the Asian turtle markets resulted in a depletion of wild Asian turtle populations [8], [9], [10], and in turn, led to an increased demand for imported species from the United States of America (US) [11], [12]. As wild populations of large turtle species (e.g., green sea turtle and alligator snapping turtle) declined due to over harvest, commercial trappers focused on smaller non-listed species [13]. In turn, turtle farming has become a booming aquaculture business in the southeastern US, especially Louisiana, in a response to meet these demands [14]. However, even with extensive farming operations, the harvest pressures on wild turtle populations remain high [15], [16].

**Figure 1:**
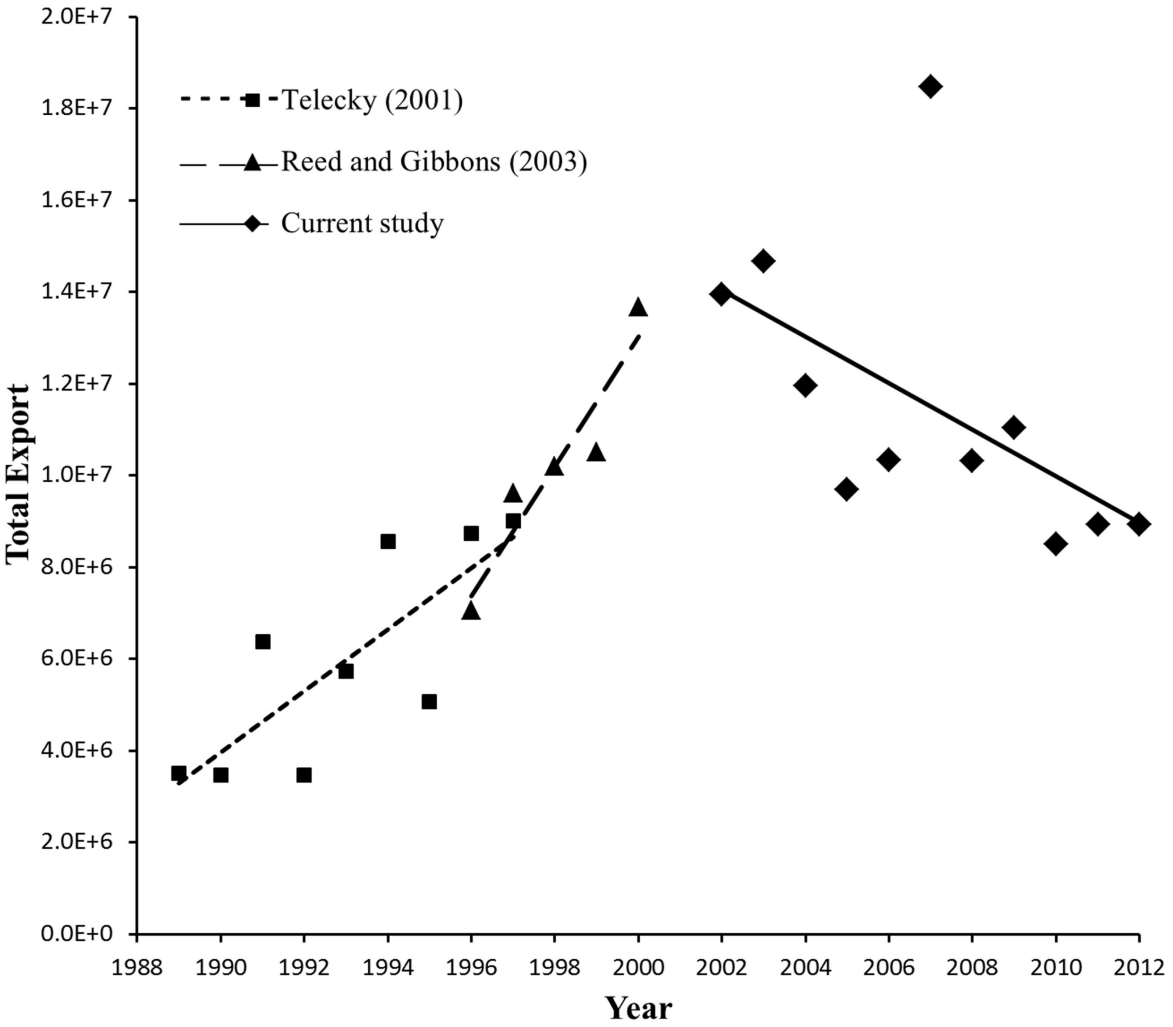
Yearly number of individual turtle exports (y-axis) from the United States as reported by Telecky (2001) [17], Reed and Gibbons (2003) [15], and what we reported for purposes of this study, including the linear trend lines. The first two studies show trending exponential increase in exports from 1989–2000 while our study shows the overall decrease for the period from 2002–2012. However, the magnitude of exports remained high (within millions) with significant increase in exports in 2007 (residual standard deviation 2.5 times higher than the average residual standard deviation), the year when the implementation of the new harvest regimes began in the southeast US.

Turtle exports from the US have increased in recent decades. Telecky (2001) [17] reported the number of exported native and non-native turtle and tortoise species rose by 257% between 1989 and 1997, from 3,485,136 to 8,990,699 individuals per year, respectively. Reed and Gibbons (2003) [15] specifically examined US native turtles and found an increase in turtle exportation, from 7,044,951 turtles exported in 1996 to 13,661,976 individuals by 2000, effectively doubling the exports within a 5 year period (Fig. 1). Ceballos and Fitzgerald (2004) [18] reported an average increase of approximately 18,000 turtles per year between 1995 and 2000. Asian countries were the major importers and the top species exported belonged to three genera of common freshwater turtle species: *Trachemys*, *Chrysemys*, and *Pseudemys*.

Large adults, females in particular, are the most valuable on the meat market and therefore a primary target of commercial trappers [19]. The adult life stage is also the most sensitive to harvest [20], [21], [22]. Research has shown harvest pressures can cause population declines in some of the most common freshwater turtle species [23]. In the US, harvest pressure has been most noticeable in the southeastern US, an ecoregion of “further conservation consideration” for freshwater turtles [24]. Scientists raised a concern for wild turtle populations and warned that the magnitude of take exceeds sustainable levels [25], [15]. The Center for Biological Diversity, with a coalition of more than 20 conservation and health groups, took action in 2007 by submitting regulatory petitions to the states of the southeast to end commercial harvest of freshwater turtles [26]. Contemporaneously, several states in the southeast US increased their restrictions on commercial take of non-listed or previously unprotected wild turtle populations.

While individual state laws control the harvest of non-threatened species, the US Fish and Wildlife Service Office of Law Enforcement (USFWS) is in charge of overviewing the export shipments. Definitions were developed by the Convention on International Trade in Endangered Species (CITES) and used by the USFWS in all states to identify and record the harvest source of individual animals or shipments. Potential sources include wild, captive bred, farmed, ranched, or turtles of unknown source; our interpretation of source definitions can be found in Table S1. However, these definitions can be vague and unclear. For example, farmed turtles are defined as being born in captivity yet farmed individual can also represent F1+ generations of wild caught turtles. Ranched individuals can be defined as wild caught but reared in a controlled environment. There are no guidelines regarding the time in captivity required for wild caught turtles to produce “captive-bred” offspring or time in captivity before wild caught turtles can be sold as “ranched” turtles. The magnitude of these discrepancies, if any, is impossible to track. According to the source, a working group in CITES has been reviewing the source code definitions for several years, but a clear conclusion has not been reached yet.

Evaluation of turtle exports remain seldom reported in the peer reviewed literature, especially the trends in more recent years subsequent to wild harvest regulations in production states. Our goal here was to analyze the magnitude of turtle exports from the US between 2002 and 2012, the period that includes the years prior to and after the implementation of commercial harvest regulations among the states in the southeastern US. We sought to 1) quantify and examine trends in the volume of live freshwater turtle exports from 2002–2012, 2) characterize the exports in terms of species, ports of export, and sources, specifically focusing on the relative magnitude of exports from wild caught individuals, and 3) review the laws in the Southeast US regarding commercial harvest and examine the possible effects they might have on the exports. We were interested in determining if closed markets influenced the magnitude of trade from adjacent exporting ports. That is, whether strict harvest regulations in one state affected the exports not only in that state, but also the surrounding states. We acknowledge that the analysis excludes any domestic trade and that the total trade numbers are much larger, but we consider exports to be a sufficient proxy of the general trends for this commercial trade and its potential impacts to native populations of freshwater turtles in the Southeast US.

## Methods

### Data

The USFWS maintains the records of all the shipments of turtles in and out of the US in the Law Enforcement Management Information System (LEMIS) database. These records can be accessed by making a request to USFWS based on the Freedom of Information Act. We queried the records of turtle shipments out of the US between 2002 and 2012. Each shipment record contains the species being shipped, the source (wild caught, captive bred, ranched, farmed, or turtles of an unknown source), description (live specimens, or bodily remains), units (number or mass of shipment in kg), purpose of trade (commercial, scientific, captive propagation purposes etc.), and the port of export. Species were recorded by the four letter codes, which were often incomplete and only reported the genus; therefore we analyzed the data at the generic level. In addition, the LEMIS data only reports the ports of export, and thus not necessarily the state the individuals were collected or originated. The LEMIS data is the only resource available that provides detailed description of export shipments and therefore the best available data from which to conduct analyses and evaluations. We also included data from Louisiana Agriculture Center [27] that keeps records of total production of freshwater turtles under farmed operations. We used this data to corroborate turtle farm production with turtle exports from this state.

We reviewed the commercial harvest management policies of freshwater turtles in the nine states of the Southeast US: Alabama, Arkansas, Florida, Georgia, Louisiana, Mississippi, Oklahoma, South Carolina, and Texas. The Association of Fish and Wildlife Agencies has assembled the state laws regarding amphibians and reptiles in a State of the Union report, last updated in December 2011 [28]. We also include regulations implemented after 2011.

### Analyses

From the LEMIS database, we sought only the exports for commercial purposes, as the shipments for scientific and conservation purposes were not our primary interest here. We only focused on the number of exported live, native, freshwater species. We summarized the total yearly exports and used least squares simple linear regression to investigate relationships between year and total amount of export [29]. We used F-tests to conduct hypothesis tests on regression coefficients, inferring significance at α = 0.05. We then classified the exports by each state. For the top four exporting states, we used a model selection approach by conducting a likelihood ratio test for two mixed effects models treating years as fixed and states as random factors. We tested the intercept only model versus the intercept and slope model. A small p-value (<0.05) indicates significant differences between the two models and the model including intercept and slope is preferred while a large p-value indicates no significant results and the model including intercept only is chosen. In the same manner, we then tested the winning model versus the same model with lag 1 temporal autocorrelation factor. For the winning model we estimated significance of the parameters. In addition, for the top four exporting states, we: 1) partitioned the taxa being exported by genus, and 2) partitioned the exports by source. For these analyses, we estimated regression coefficients across the states in order to infer any significant trends. Further, for those genera representing the majority of exported individuals, we examined the proportion of wild caught turtles. We performed statistical analyses using R version 2.10.1 (The R Foundation for Statistical Computing, Vienna, Austria). Once the data were analyzed, we linked the export trends with the management regimes implemented in each state.

## Results

### Overall Trends

Between 2002 and 2012, a total of 126,600,529 individual freshwater turtles were exported from the US. Based on the marginally significant simple linear regression (F = 3.91; df = 1,9; p = 0.08), the number of exported turtles decreased on average 500,000 turtles per year over the 11 year period. However, in 2007, residual standard deviation was 2.5 times higher than the average residual standard deviation. In 2007, there was a 79% increase (18,457,520 individual turtles) compared to 2006 (Fig. 1). Overall, 53% were commercially bred, 28% were classified as farmed or ranched, and 19% were classified as wild caught individuals. When we partitioned the total exports by source, the number of captive bred exports declined after 2007 while wild caught exports increased after 2009 (Fig. S1).

### Exported Taxa

**Figure 2:**
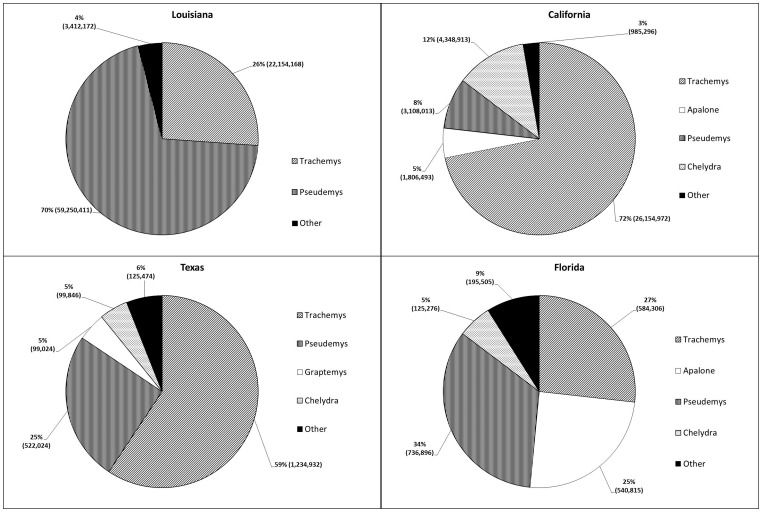
Percentage of different genera of native freshwater turtles exported from Louisiana, California, Texas, and Florida from 2002–2012. Majority of exports belonged to the genera *Pseudemys* and *Trachemys*.

The following genera were exported: Apalone, Chelydra, Chrysemys, Clemmys, Deirochelys, Emydoidea, Graptemys, Kinosternon, Macroclemys, Malaclemys, Pseudemys, Sternotherus, Terrapene, and Trachemys. Combined, Pseudemys and Trachemys represented between 61% (Florida; 1,321,202 individual turtles) and 96% (Louisiana; 81,404,579 individual turtles) of all species traded from the top four exporting states (Fig. 2). Chelydra consisted of 12% (4,248,913 individuals) of the exports from California, 5% (99,846 individuals) of the exports from Texas, and 5% (125,276 individuals) of the exports from Florida. Apalone consisted of 25% (540,815 individuals) of all exports from Florida and 5% (99,024 individuals) from Texas. For the top four exported genera (Apalone, Chelydra, Pseudemys, and Trachemys), regression coefficients showed significant increase in traded Trachemys in Louisiana (p = 0.02) and significant decrease in traded Trachemys in California (p<0.01). Traded Apalone significantly increased in Florida and California (p<0.01) while traded Chelydra increased in Louisiana and California (p<0.01).

Considering the high magnitude of exports from Louisiana and California, it is worth reporting the totals among all exported genera (Table 1).

**Table 1:** Total number of exported freshwater turtles from the top two exporting states from 2002–2012 sorted by the genus.

*Genus*	Louisiana	California
*Pseudemys*	59,240,411	3,108,013
*Trachemys*	22,154,168	26,154,972
*Graptemys*	1,822,122	132,684
*Chelydra*	664,912	4,348,913
*Sternotherus*	325,238	337,397
*Apalone*	300,679	1,806,493
*Chrysemys*	217,519	132,684
*Macroclemys*	73,602	233,916

### Exporting States

**Figure 3:**
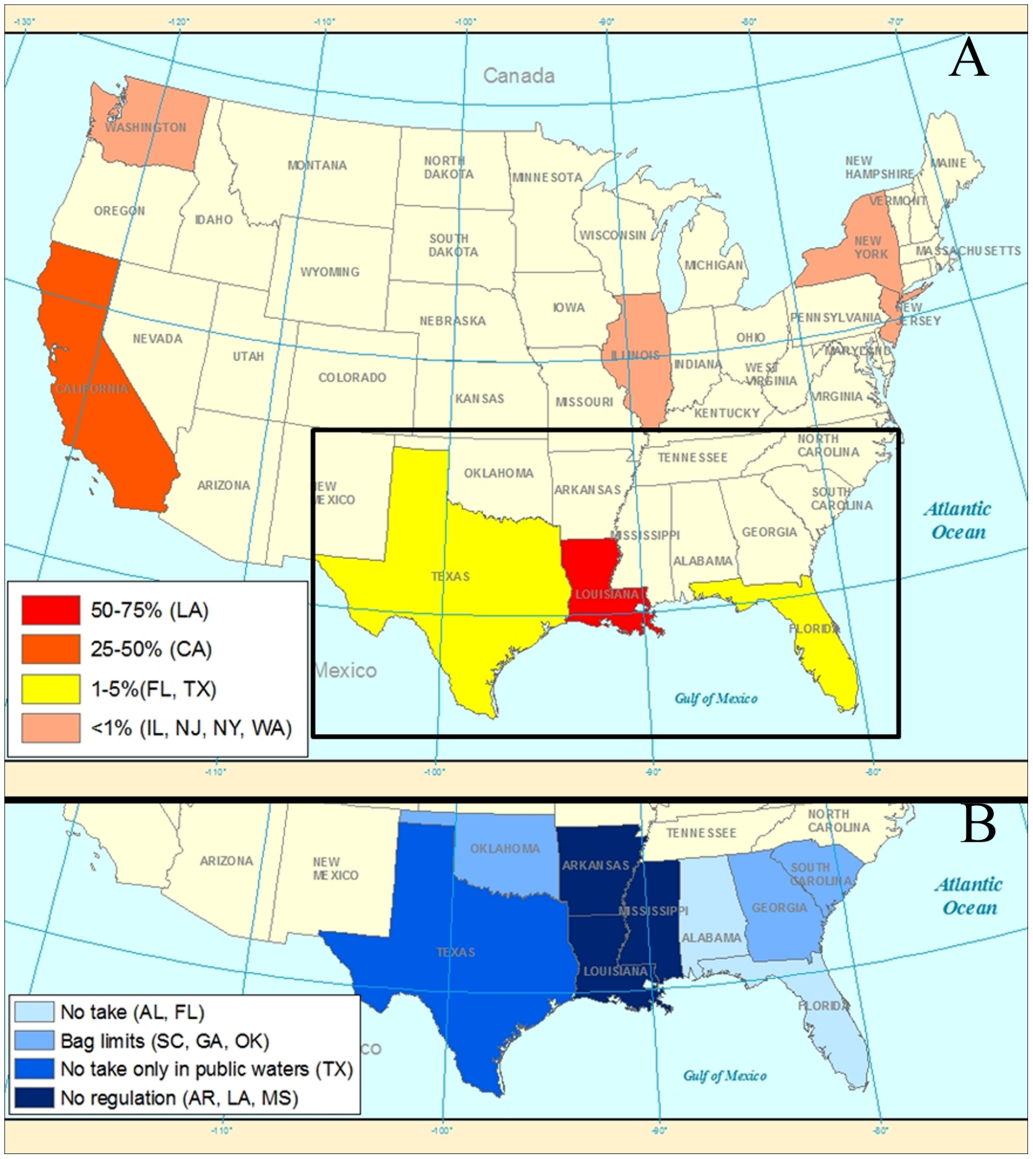
Proportion of freshwater turtles exported from the US in the period from 2002–2012, partitioned by the exporting state (A), and an overview of state regulations in the southeast US (B), the region with the further conservation concern regarding freshwater turtles. Four states (Louisiana, California, Texas, and Florida) account for 96% of all exports. On the other hand, Louisiana, Arkansas, and Mississippi still allow unlimited take while Alabama and Florida banned commercial harvest from all water bodies and therefore represent two states with the strictest laws regarding commercial turtle harvest.

**Figure 4:**
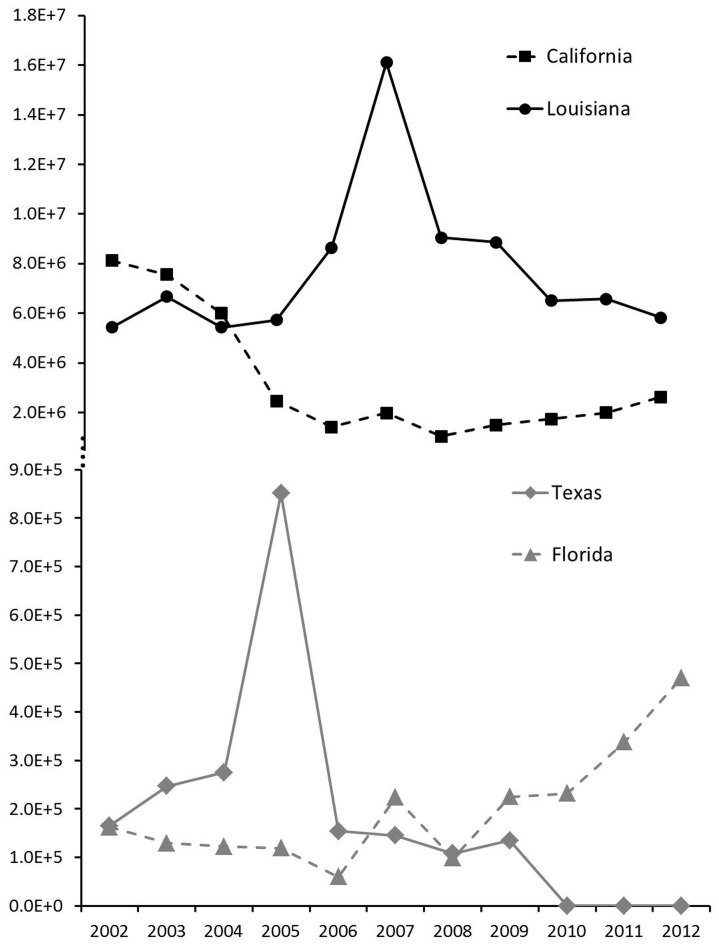
Number of native freshwater turtles (y-axis) exported from Louisiana, California, Texas, and Florida from 2002–2012 (x-axis). Louisiana and California accounted for 96% of the overall export from the US. Texas and Florida accounted for 2% each of the overall export from the US.

We broke the dataset down into the number of exports in each state. Turtles were exported out of California, Florida, Georgia, Hawaii, Illinois, Louisiana, New Jersey, New York, Texas, and Washington (Fig. 3A). Overall, Louisiana and California accounted for 96% of the exports (67% and 29% respectively), followed by Texas and Florida (2% each; Fig. 4). The model containing intercept only was superior to the intercept slope model (p = 0.28) and the model containing lag 1 autocorrelation factor was superior to the intercept only model (p<0.01). The winning model showed no significant trend in exports over the 11 year period among the states (p = 0.49), but the correlation parameter estimate of 0.75 indicated high temporal autocorrelation within the dataset.

On average, 7,710,614 and 3,309,426 turtles/year were exported from Louisiana and California, respectively. Across all years, Louisiana exported the most turtles in 2007 (16,105,077 turtles). Exports out of California gradually decreased between 2002 and 2005, and throughout the next seven years, approximately 2 million turtles were exported per year. Although Texas and Florida account for a small proportion of total exports, there was an increase in exports from Texas in 2005 (850,940 individual turtles exported) and an increase in exports from Florida in 2007 (223,895 individual turtles). In all cases except Florida, the number of exports decreased dramatically after these peaks and continued to slowly decrease. However, exports out of Florida have steadily increased since 2008.

### Turtle Sources among States

**Figure 5:**
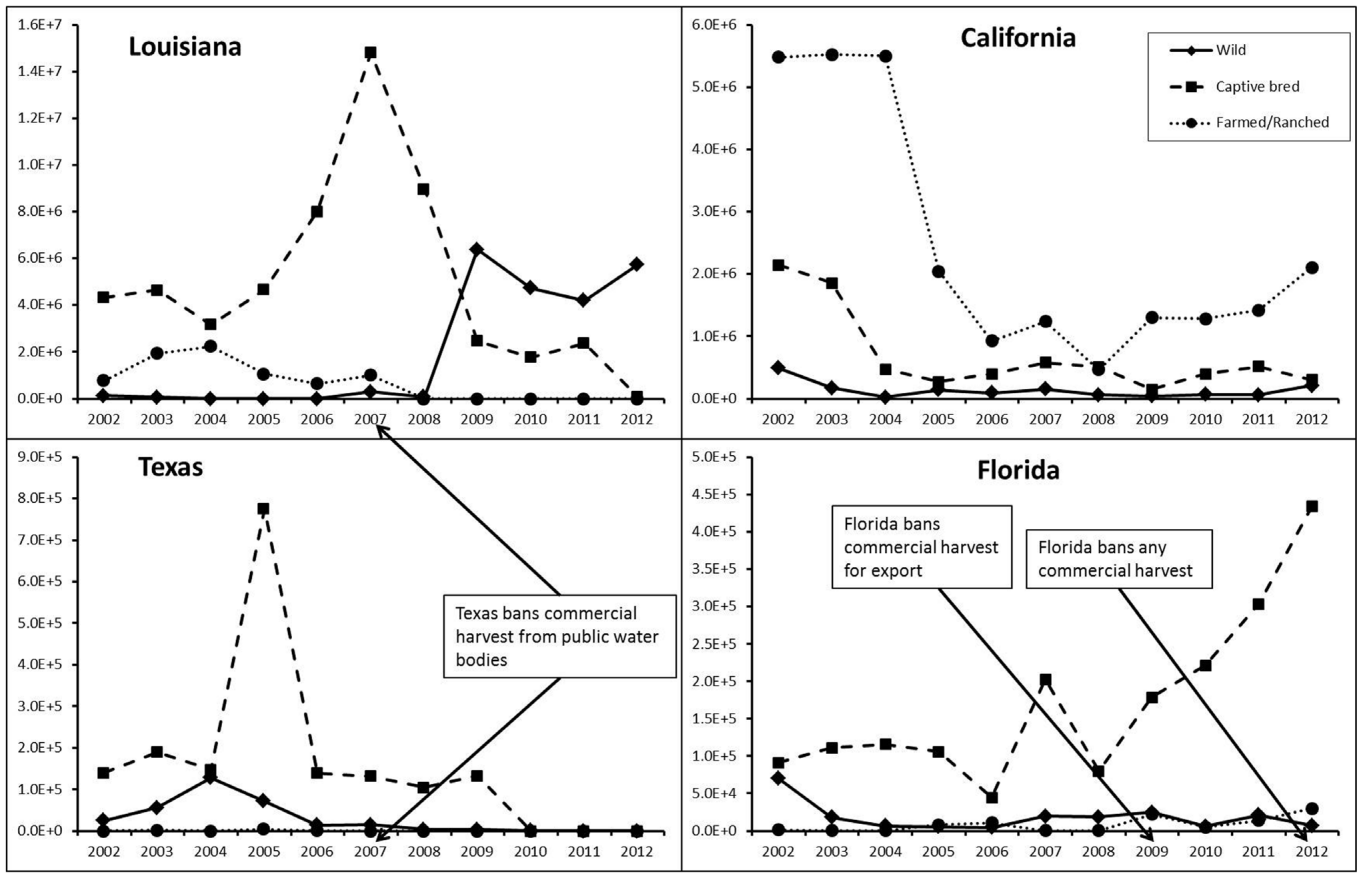
Numbers of native freshwater turtles (y-axis) exported from Louisiana, California, Texas, and Florida from 2002–2012 (x-axis), separated by sources.

In Louisiana, Florida, and Texas captive bred individuals comprised the majority of exports (65%, 86%, and 85%, respectively) while in California, farmed/ranched individuals composed the majority of exports (75%; Fig. 5). Regression coefficients showed significant increase in exported wild individuals in Louisiana (p<0.01) and a significant decrease in exported farmed/ranched individuals in Louisiana and California (p = 0.01 and p<0.01, respectively).

For each of the top four states, any change in exports among the years corresponds with the change in exports of captive bred individuals. Comparatively, wild caught turtles were exported in much smaller quantities than captive bred individuals. In Louisiana, however, the number of exported wild caught turtles increased from 80,050 in 2008 to 6,386,030 in 2009 and remained high the following three years and exceeded the number of captive bred turtles exported.

### Wild Turtle Exports

**Figure 6:**
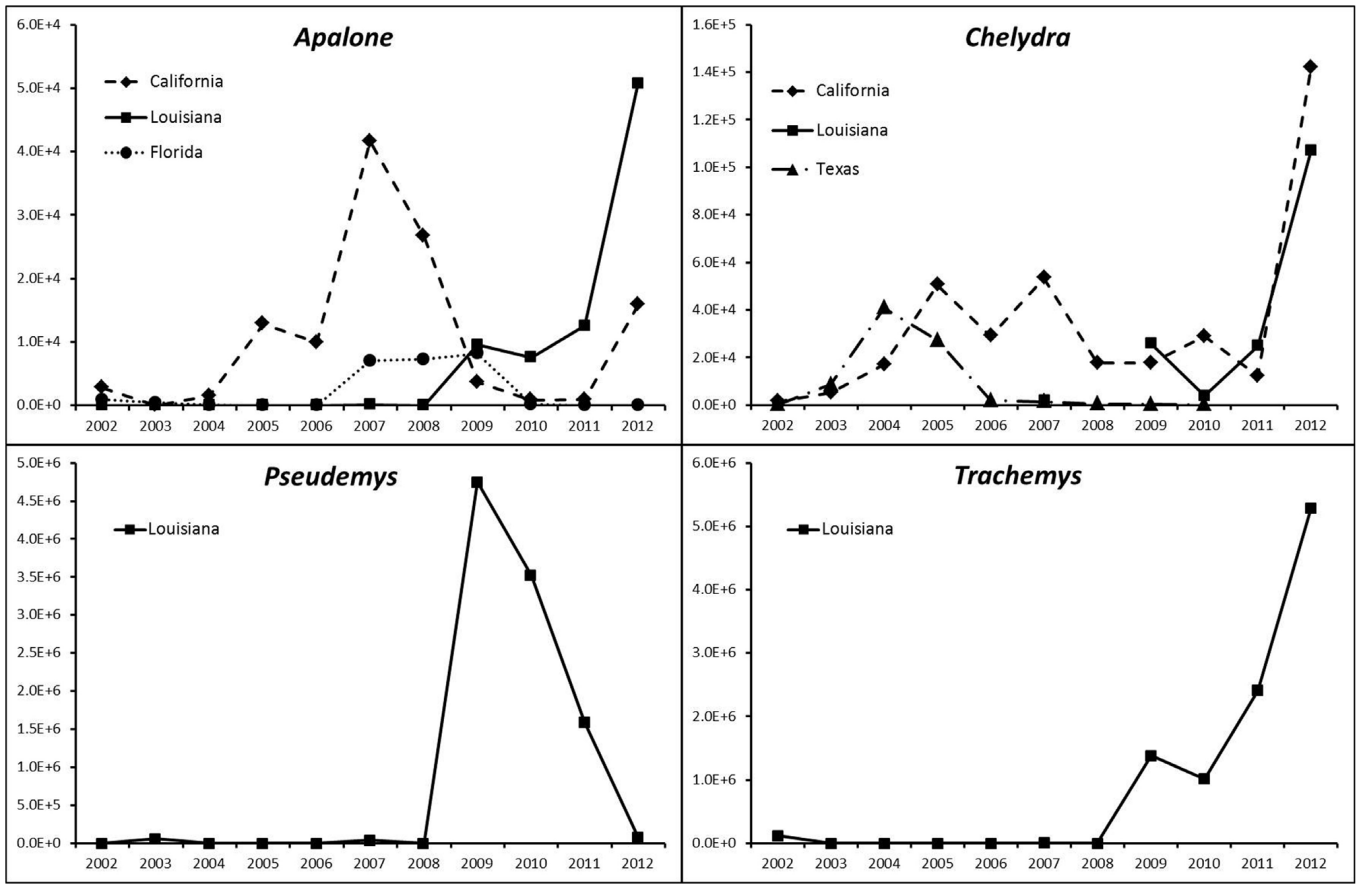
Numbers of wild caught *Apalone*, *Chelydra*, *Pseudemys*, and *Trachemys* (y-axis) shipped from the major exporting states from 2002–2012 (x-axis). Louisiana was a major exporter of *Pseudemys* and *Trachemys* while Louisiana and California were the major exporters of *Apalone* and *Chelydra*.

Louisiana reportedly exported the most wild caught individuals of any state (Fig. 5). The number of wild exports significantly increased for *Apalone*, *Chelydra*, *Pseudemys*, and *Trachemys* (p<0.01) while California increased exports of wild *Chelydra* (p<0.01). Most wild caught *Trachemys* were exported from Louisiana. However, prior to 2009, the number of exported *Trachemys* from Louisiana was trivial in contrast to 2009 when over a million wild caught turtles were exported. Since then, the number of turtles exported steadily rose, achieving 5,288,482 individuals in 2012. Louisiana was also a primary exporter of wild *Pseudemys*, but like *Trachemys* exports were negligible until 2009. In 2009, over four million *Pseudemys* were exported yet the numbers gradually declined as the number of *Trachemys* exported increased (Fig. 6). Since the definition of wild caught can include F1 hatchlings from wild caught brood stock, we also gathered information from Louisiana Department of Agriculture and examined the number of captive bred turtles over the same period. We noticed the number produced after 2009 closely matched the captive bred reported in the LEMIS data (Fig. S2). This observation suggests the wild exports out of Louisiana were most likely turtles directly harvested from wild populations.

### Harvest Management Regimes

As a response to submitted petitions to ban commercial harvest, six out of nine states in the Southeast US provide different levels of protection (Fig. 3B). Alabama and Florida now have the strictest laws on commercial take (Table 2), prohibiting any commercial harvest from public and private water bodies. While Alabama implemented its laws for the first time in 2012, Florida began implementing the new regulations three years prior. In 2009, Florida banned commercial harvest for export, but turtle farmers were allowed to continue harvesting essentially unlimited numbers of wild turtles for aquaculture with appropriate permits [28]. These permits expired in April of 2012, with Florida then implementing a prohibition of commercial harvest. After Florida banned commercial harvest for export, exports of captive bred individuals increased, but we observed no apparent change in wild caught turtle exports after the ban (Fig. 5) Texas provided some level of harvest management beginning in 2007 with a ban of commercial harvest from public water bodies. By 2010, zero wild caught turtles were exported out of Texas. South Carolina established daily and annual bag limits in 2009 while Georgia and Oklahoma established daily and bag limits in 2012.

**Table 2:** Summarized commercial collection limits within each state in the southeast US and its legal and regulatory provisions.

State	Regulation	Daily/annual bag	Size restrictions	Citation
Alabama	No take			[33]
Arkansas	Open season	Unlimited	Any	[28]
Florida	No take			[28]
Georgia	Open season	10 turtles/day	Any	[34]
Louisiana	Open season	Unlimited	Any	[28]
Mississippi	Open season	Unlimited	Snapping turtles	[28]
Oklahoma	Open season	6 turtles/day	Any	[28]
South Carolina	Open season	10 turtles/vehicle; 20 turtles/year	Any	[28]
Texas	Private waters only	Unlimited	Any	[35]

The remaining three states, Arkansas, Louisiana, and Mississippi permit essentially unlimited take. Until 2009, the vast majority of exports from Louisiana were captive bred individuals. While the number of captive bred exported turtles slowly decreased after 2007, export of wild caught turtles from Louisiana has since steadily increased. According to the LEMIS and Louisiana Department of Agriculture data, wild caught turtles now make up the largest portion of the total exports from Louisiana (Fig. 5). The Louisiana Department of Agriculture (2012) reported that competition among turtle farms eventually led to a decrease in the number of total farms in the state. It is possible that this decline in turtle farms could account for the decrease in farmed exports out of Louisiana.

## Discussion

Turtles are an important part of the aquatic ecosystems. While relatively little work has been published directly seeking their contributions to food webs or ecosystem function, their contribution to these functions is important [30]. Over harvest can cause population declines in even the most common species, and subsequently cause changes in energy flow, nutrient cycling, and food web structure. In the last three decades, the US became a major turtle exporter that has supplied the otherwise depleted Asian food markets [15]. In response to concerns raised by researchers and biodiversity groups, alongside increased awareness of the magnitude of this harvest by state regulatory agencies, several states in the southeast have banned commercial harvest of freshwater turtles within the past five years.

We summarized freshwater turtle exports from the US across the past 11 years and found that the magnitude of export has remained high when compared with previous reports [17], [15] (Fig. 1). However, the overall exported quantity has been decreasing, with marginal statistical support (Fig. 1). Also, the proportion of wild caught individuals (19%) was less than previously reported (34%) [15]. The decrease could potentially be a positive outcome from the new management regimes. However, if the harvest effort has remained the same over the past 11 years, this may alternatively, indicate a decline in freshwater turtle numbers as fewer individuals are found to bring to market.

The vast majority of exports belonged to common species of the genera: *Trachemys*, *Pseudemys*, *Chelydra*, and *Apalone*. Although these are not protected under CITES, research has shown that populations of some of the most utilized taxa, such as *Trachemys scripta elegans*, can become significantly depleted under intense harvest pressures, taking these populations decades to recover [23]. Although statistical analysis failed to detect significant differences in the exportation trends, we cannot ignore states with harvest regulations decreased wild turtle exports and states without harvest regulations increased wild turtle exports.

We did observe short term responses to regulation within states that have emplaced changes to commercial utilization of turtles. We are particularly referring to Texas, where, at least according to LEMIS, commercial harvest is no longer occurring. After Florida first implemented harvest regimes in 2009, the state continued to export wild caught individuals. It is of our further interest to continue monitoring exports from Florida especially after the complete harvest ban in 2012. The results also indicate that these regimes could have potential negative effects on wild turtle harvest in the surrounding states, potentially placing extreme harvest pressure on Louisiana’s wild turtle populations. Ultimately, the lack of clear data describing the actual origin of exported turtles enables more than one interpretation of the data.

First, commercial trappers from the states with new harvest management regimes may have simply shifted their exportation ports to nearby states (e.g., Louisiana). Large scale turtle harvest is organized as a pyramid scheme including trappers, middlemen, and dealers. Turtle dealers usually have an interstate network of several hundred employees and are capable of exporting hundreds of thousands of turtles a year (Texas Parks and Wildlife Department, pers. comm.). It is less likely that harvest regulations in one state could impact this business network. There are currently no laws in place to declare the state of origin of wild caught turtles, aside from obtaining state harvesting permits, nor are there any interstate trade regulations for non-CITES listed species. Therefore, perhaps wild caught turtles from the surrounding regulated states are now being exported through Louisiana. This alternative hypothesis is further supported by the numbers of exported turtles out of California, a state with only one native freshwater turtle species, the western pond turtle *Actinemys marmorata* [31].

California has remained a top exporter of turtles, matching the magnitude of turtles shipped from Louisiana. The majority of exported turtles from California are farmed or ranched individuals. We were not able to find information on extensive turtle farming operations in California, certainly not at the scale of the Louisiana Turtle Farmer’s association (California Aquaculture Association, pers. comm.). Therefore, we are confident to say that exported *Apalone*, *Chelydra*, and *Trachemys* did not originate from California, and this makes any assessment of the origin for these California exports difficult to track. In 2012, an owner of a turtle aquaculture facility in Florida was convicted of illegally marking wild caught turtles as captive bred and attempting to export these turtles out of Los Angeles International Airport [32]. Understanding the domestic origin of these shipments or the domestic origin of the turtles themselves is crucial to our understanding of the commercial trade of freshwater turtles in the US, and the lacking public information at hand is worrisome.

### Management Recommendations

To improve our understanding of temporal trends in turtle exports, we recommend establishing better guidelines for labeling the sources of exported turtles. Clear and reliable standards set for all turtle exports from the US would include required confirmation of captive bred versus wild animals and reporting of originating states, not just the port of export. Moreover, animals that are directly taken from the wild and being exported immediately (e.g., the same year) must be clearly separated from any other category. In addition, we recommend developing a new category for the shipments that would classify exported turtles as hatchlings (juveniles less than 1 year since hatching) or adult turtles. A new category would provide better understanding of whether the turtles are being exported for food markets (adult turtles) or possibly pet markets (hatchlings). The LEMIS database had previously included values per shipment which did enable inference as to whether the shipment includes low priced hatchlings that are most likely coming from farming operations or higher price adult turtles. We strongly recommend re-establishing this category. With these improvements of shipment recording, LEMIS database could be more useful in evaluating the trends and consequences of harvest regimes.

Based on our findings, we have shown that the strict regulations in one state may well have a negative harvesting influence in surrounding states with and without regulatory laws. The reasons are twofold. Primarily, strict regulations in one state could act to drive increased harvest in unregulated states. Secondarily, harvest in regulated states could illegally continue if turtles can be exported from a neighboring state. Arkansas, Louisiana, Mississippi, and Georgia are in the center of the freshwater biodiversity hotspot for the US and all still lack strict regulatory legislation (i.e., prohibition of harvest from the wild) on the commercial harvest of the most utilized taxa. Regulating commercial harvest by implementing a ban on the most sensitive life stages (adults, specifically adult females) in these states, especially exporting states (e.g., Louisiana) would also strengthen the laws previously established in surrounding states. The simple requirement of legal certification and documentation by the shipping agent that all turtles exported from these unregulated states, originated there, would enable better control and law enforcement during shipment operations. For example, under current circumstances, a turtle dealer residing in Louisiana could be harvesting turtles from Texas or Florida but reporting them as Louisiana turtles. There are currently no requirements to verify the origin or category of turtles exported.

States of the Southeast US implement a variety of harvest regulations: complete ban, establishing season and bag limits, or complete ban in certain areas (e.g., public waters) and no season or bag limits for the utilized taxa in the rest (e.g., private waters). As a complete ban is certainly the strictest possible law and the hardest to pass, we recommend establishing seasons and bag limits as a first step toward protection. Within a state, we do not recommend protection of certain areas while leaving the other areas open to unlimited harvest because there is no supporting evidence that protected populations would replenish the harvested areas.

## Supporting Information

Figure S1 Total number of exported turtles (y-axis) from 2002–2012 (x-axis) partitioned by the source of turtles.
(PDF)

Figure S2 Total production of freshwater turtles (y-axis) on Louisiana farms from 2002–2012 (y-axis).
(PDF)

Table S1 Definitions of the sources of exported freshwater turtles used by the USFWS during the inspection of shipments.
(PDF)
