# Supplementary material for: Magnitude of the Freshwater Turtle Exports from the US: Long Term Trends and Early Effects of Newly Implemented Harvest Management Regimes
Source: PLoS One. 2014 Jan 27;9(1):e86478. doi: 10.1371/journal.pone.0086478 (PMC3903576; doi:10.1371/journal.pone.0086478)
Supplement: Figure S1 — Total number of exported turtles (y-axis) from 2002–2012 (x-axis) partitioned by the source of turtles. (PDF) [file pone.0086478.s001.pdf]

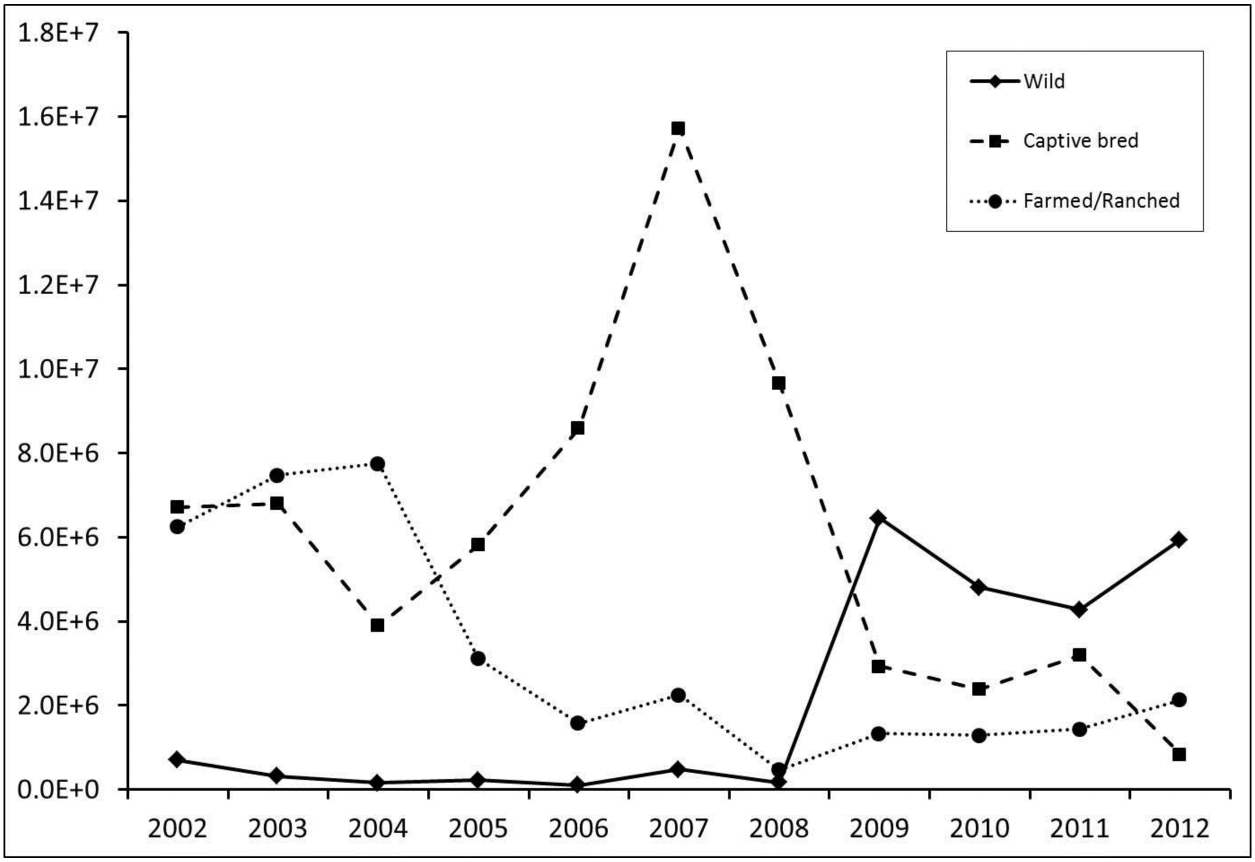

Supplemental Fig. S1. Total number of exported turtles (y-axis) from 2002-2012 (x-axis) partitioned by the source of turtles.
