# Supplementary material for: Magnitude of the Freshwater Turtle Exports from the US: Long Term Trends and Early Effects of Newly Implemented Harvest Management Regimes
Source: PLoS One. 2014 Jan 27;9(1):e86478. doi: 10.1371/journal.pone.0086478 (PMC3903576; doi:10.1371/journal.pone.0086478)
Supplement: Table S1 — Definitions of the sources of exported freshwater turtles used by the USFWS during the inspection of shipments. (PDF) [file pone.0086478.s003.pdf]

Supplemental Table S1. Definitions of the sources of exported freshwater turtles used by the USFWS during the inspection of shipments.

Source of specimen	Description
Bred-in-captivity	The specimen was born to parents that either mated or transferred gametes in a controlled environment
F1 (farmed)	Born in captivity to wild-caught parents but are not considered as captive bred under CITES
Ranched	Directly removed from the wild and reared in a controlled environment or are progeny from gravid females captured from the wild
Wild	Specimen taken from the wild or specimen born in captivity from an egg collected in the wild
